# Prognostic Models for Patients With Gleason Score 9 Prostate Cancer: A Population-Based Study

**DOI:** 10.3389/fonc.2021.633312

**Published:** 2021-04-26

**Authors:** Jianhui Qiu, Desheng Cai, Zixin Wang, Jingcheng Zhou, Yanqing Gong, Lin Cai, Kan Gong

**Affiliations:** ^1^Department of Urology, Peking University First Hospital, Beijing, China; ^2^Institute of Urology, Peking University, Beijing, China; ^3^National Urological Cancer Center, Beijing, China

**Keywords:** Gleason score, prostate cancer, all cause mortality, cancer specific mortality, Gleason pattern

## Abstract

**Purpose:** Gleason score (GS) system is one of the most widely used histological grading methods for prostate cancer (PCa) all over the world. GS can be obtained by adding the primary Gleason pattern (GP) and secondary GP. Different proportions of GP 4 and GP 5 in prostate specimens can both lead to GS 9. In this study, we explored whether GP 5 + 4 or GP 4 + 5 was associated with different prognoses among patients with GS 9 PCa.

**Materials and methods:** A retrospective population-based study was conducted on 10,124 subjects diagnosed with GS 9 PCa between 2004 and 2009 from the Surveillance, Epidemiology, and End Results program. A 1:1 propensity-score matching (PSM) was performed to balance the baseline characteristics between the GP 4 + 5 and 5 + 4 groups and to compare the prognoses between the two groups. Cox regression analysis and Fine-Gray competing risk regression models were adopted to screen the covariates significantly associated with all-cause mortality (ACM) and cancer-specific mortality (CAM).

**Results:** GP 5 + 4 was associated with higher risks of ACM and CSM before or after PSM than GP 4 + 5. In the original cohort, there were eight independent predictors for ACM, which were age at diagnosis, race, AJCC NM stage, PSA levels, treatments, GP, and marital status, confirmed by the Cox analysis; and nine independent predictors for CSM, which were age at diagnosis, race, AJCC TNM stage, PSA levels, treatments, GP, and marital status, confirmed by the competing-risk model.

**Conclusion:** GP 5 + 4 was associated with a poorer overall survival and cancer-specific survival compared with GP 4 + 5.

## Introduction

Prostate cancer (PCa) was one of the leading genitourinary neoplasms in males all over the world, although the incidence rate of PCa differs slightly with the changes in the area and race (1, 2). The Gleason score (GS) system, first described by Gleason (3), is generally believed to be an important prognostic predictor. GS 9–10 is associated with poorer outcomes than GS 8 (4, 5). The current clinical disease staging methods regarded patients with GS 9–10 PCa as an independent group from patients with other GS and assumed that patients with GS 9–10 PCa undergo similar risks of recurrence, metastasis, and mortality (6). However, different Gleason patterns (GP), GP 4 + 5 and 5 + 4, can both lead to GS 9. It has been reported that an increased proportion of Gleason pattern 5 has a trend to be associated with adverse outcomes (7). Several previous studies have proved differences in the prognoses between patients with GP 4 + 5 and 5 + 4 PCa (8–10). Here, we perform a study with a larger survey sample in the individual level to investigate the differences in the overall survival (OS) and cancer-specific survival (CSS) between the two GPs.

Deaths from non-cancer causes act as competing events of cancer-specific mortality (CSM) (11). Therefore, rather than the Kaplan-Meier method, Fine-Gray competing-risk regression models were adopted in this study to analyze the CSS in the presence of competing events.

Surveillance, Epidemiology, and End Results (SEER) database is an authoritative source of population-based data in the U.S., which records cancer stage at the time of diagnosis and patient survival information (12). In this study, the follow-up information on GS 9 PCa was extracted from the SEER database.

## Materials and Methods

### Study Population

**Figure 1:**
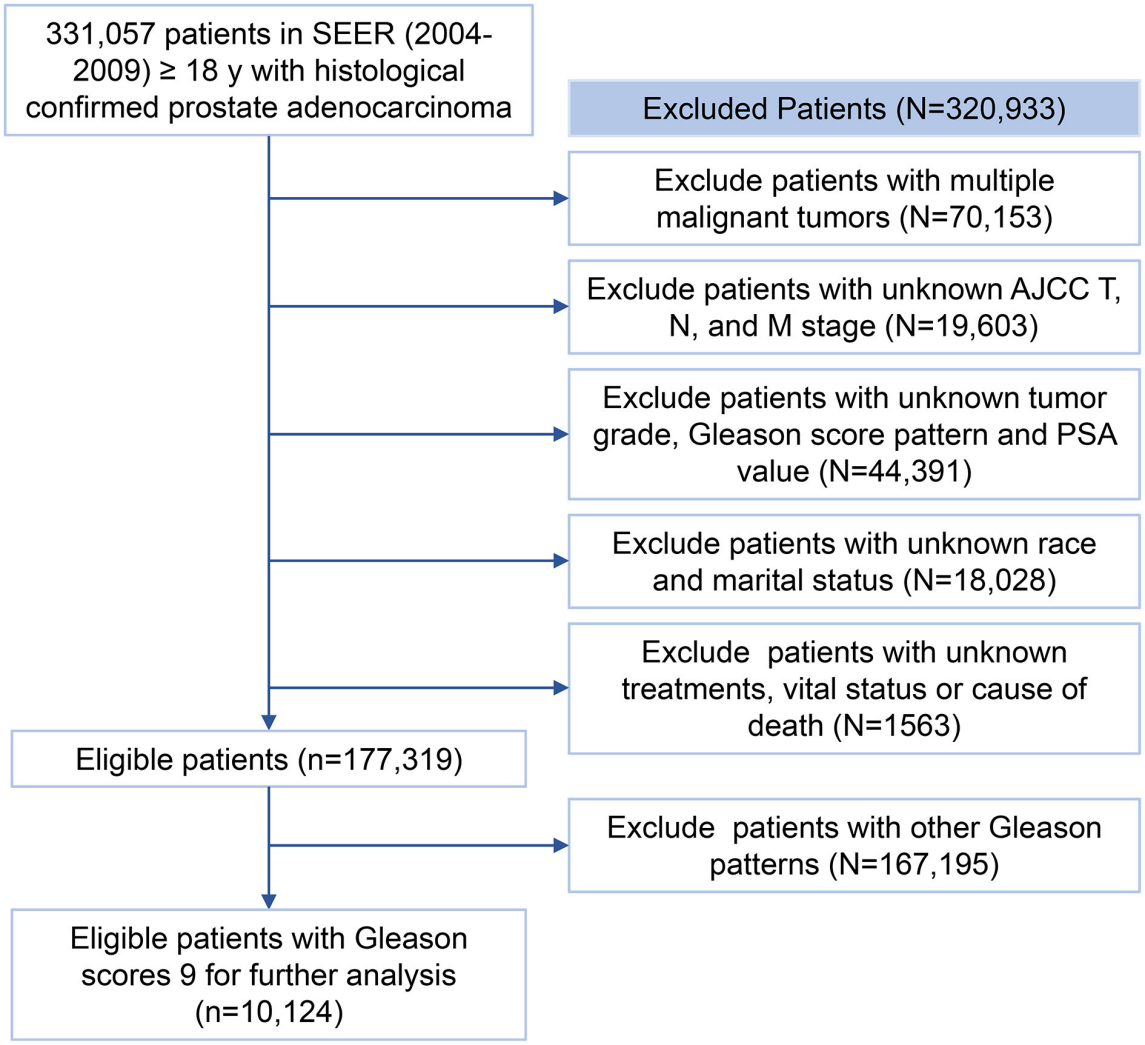
The flow chart of study participants selection. PSA, prostate-specific antigen.

The diagnosis in the SEER registry (https://seer.cancer.gov/data/) was coded by the International Classification of Disease for Oncology-3 (ICD-O-3). Patients diagnosed with PCa (topography codes: C61.9; histological code:8140: 3) between 2004 and 2009 were retrieved from the SEER registry. Subjects with one of the following conditions will be excluded: (1) with more than one primary malignant tumors; (2) AJCC TNM stage was unknown; (3) tumor grades, prostate-specific antigen (PSA) levels, and GPs were unknown; (4) with unknown marital status and ethnicity; (5) with unknown treatments or follow-up information. The screening process for the patients recorded in the SEER registry was shown in Figure 1. The entire cohort obtained after the screening was later randomly divided into a training group and a validation group in a 2:1 ratio for the establishment and validation of the prognostic models.

### Propensity Score-Matching

The multivariate logistic regression analysis was used to get the propensity scores for each subject based on the age at diagnosis, AJCC stage, AJCC TNM stage, treatments, and marital status. GP 4 + 5 and 5 + 4 groups were matched in a 1:1 ratio through a caliper width of 0.05 for the propensity score and the nearest neighbor matching.

### Study Variables

Age at diagnosis was divided into three groups, <72, 72–78, >78, using the X-tile software (version 3.6.1, Robert L Camp, Yale University). The race was categorized as black, white, and others (American Indian/Alaska Native, Asian, or Pacific Islander). The marital status was classified as married or unmarried (separated, divorced, single, or widowed, etc.). The professional quality evaluation and validation of PSA in the SEER registries confirmed precise PSA values for further analyses (13). PSA levels were divided into <10, ≥20, and ≥10& <20 ng/ml. AJCC T status was categorized as T1/2 and T3/4. According to the SEER database, the treatments to the patients were classified into four types as follows: neither surgery nor radiation, only surgery, only radiation, and both surgery and radiation.

### Statistical Analysis

Continuous variables were depicted using medians and interquartile ranges, while categorical variables were reported by proportions. The chi-square tests were used to compare the baseline categorical variables between the GP 4 + 5 and 5 + 4 groups and the Mann-Whitney U tests were used for continuous variables, such as age at diagnosis. ACM and CSM were the primary outcomes of this study according to the record of the SEER database. The Kaplan-Meier method as well as log-rank tests were used to compare the OS between groups. Univariate and multivariate Cox regression analysis models were adopted to detect factors influencing OS. Fine-Gray competing-risk regression models were used to assess the predictive factors of CSS. The forward stepwise method was used to confirm the predictive factors included in the final multivariate models.

X-tile version 3.6.1(Robert L Camp, Yale University, https://x-tile.net/) was adopted to classify the continuous variables (age at diagnosis) (14). Kaplan–Meier methods and Cox analysis were performed using the IBM SPSS Statistics version 23.0 (SPSS Inc., Chicago, IL, USA). Fine-Gray competing-risk regression and the PSM process were conducted using the Stata/SE version 15.1 (StataCorp, College Station, Texas). All statistical tests were two-sided with a *P* < 0.05 considered to be indicative of statistical significance.

## Results

### Study Population

Three hundred thirty-one thousand fifty-seven (331,057) patients diagnosed with PCa between 2004 and 2009 were collected from the SEER database. Finally, 10,124 eligible patients with PCa of GS 9 were included for further analysis as shown in Figure 1. Three thousand one hundred eighty-nine (3,189) failure events and 2,003 competition events were observed in the overall cohort.

**Figure 2:**
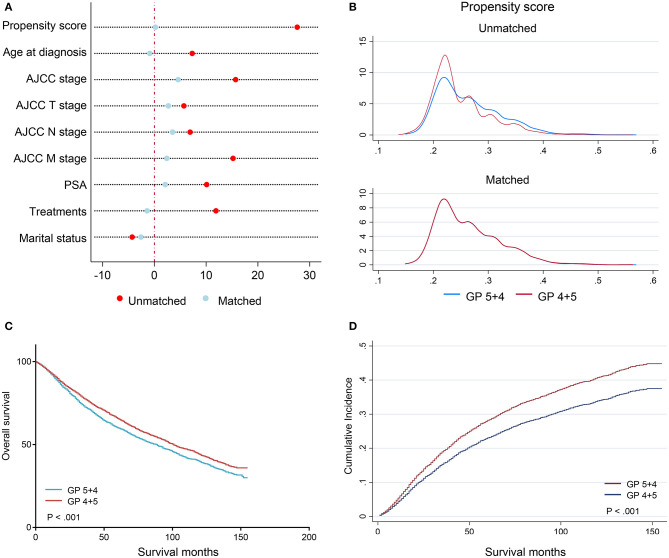
Plots after PSM in a 1:1 ratio. **(A)** the love-plot (dot chart) of the standardized percentage bias for each covariate before and after PSM; **(B)** the kernel density plot of propensity score before and after PSM; **(C)** the Kaplan–Meier curves stratified by Gleason patterns after PSM; **(D)** the CIF curves stratified by Gleason patterns. PSM, propensity score-matching.

Baseline characteristics of patients between the GP 4 + 5 and the GP 5 + 4 groups before and after PSM were shown in Table 1. In the entire cohort, 7,759 (74.7%) patients were diagnosed with GP 4 + 5 PCa and 2,565 (25.3%) with GP 5 + 4 PCa. Patients in the GP 5 + 4 group were associated with an increasing age at diagnosis (*p* = 0.002) and a higher AJCC N (*p* = 0.002) and M (*p* < 0.001) stage compared with those in the GP 4 + 5 group. After PSM, a cohort of 5,130 patients was generated, 2,565 patients in each group. The baseline characteristics were well-balanced between the two groups in the after-PSM cohort (Table 1, Figures 2A,B). One thousand eight hundred fifty-six (1,856) failure events and 1,029 competition events were recorded in the post-PSM cohort. The median follow-up period was 90 months before PSM and 86 months after PSM.

**Table 1:** Baseline characteristics of patients between the GP 4 + 5 and 5 + 4 groups before (*n* = 10,124) and after PSM (*n* = 5,130).

**Characteristics**	**Overall cohort (*****n*** **=** **10,122)**			**After PSM (*****n*** **=** **5,130)**		
	**4 + 5 group (*n* = 7,559; 74.7%)**	**5 + 4 group (*n* = 2,565; 25.3%)**	***p*-value**	**4 + 5 group (*n* = 2,565)**	**5 + 4 group (*n* = 2,565)**	***p*-value**
Follow-up period (months), median (IQR)	90 (43, 114)		NA	86 (36, 110)		NA
	92 (49, 1)	82 (32, 110)	NA	88 (40, 111)	82 (32, 110)	NA
Age at diagnosis (y), median (IQR)	68 (61, 75)	69 (62, 76)	0.002	69 (62, 76)	69 (62, 76)	0.889
**Race**, ***n*** **(%)**						
White	5,873 (77.7%)	1,997 (77.9%)	0.840	2,007 (78.2%)	1,997 (77.9%)	0.792
Black	1,091 (14.4%)	375 (14.6%)		359 (14.0%)	375 (14.6%)	
Others	595 (7.9%)	193 (7.8%)		199 (7.8%)	193 (7.5%)	
**AJCC stage**, ***n*** **(%)**						
II	4,190 (55.4%)	1,262 (49.2%)	<0.001	1,313 (51.2%)	1,262 (49.2%)	0.264
III	1,604 (21.2%)	522 (20.4%)		521 (20.3%)	522 (20.4%)	
IV	1,765 (23.3%)	781 (30.4%)		731 (28.5%)	781 (30.4%)	
**AJCC T stage**, ***n*** **(%)**						
T1	1,791 (23.7%)	616 (24.0)	<0.001	624 (24.3%)	616 (24.0%)	0.708
T2	3,260 (43.1%)	1,046 (40.8%)		1,074 (41.9%)	1,046 (40.8)	
T3	2,124 (28.1%)	689 (26.9%)		670 (26.1%)	689 (26.9%)	
T4	384 (5.1%)	214 (8.3%)		197 (7.7%)	214 (8.3%)	
**AJCC N stage**, ***n*** **(%)**						
N0	6,673 (88.3%)	2,205 (86.0%)	0.002	2,235 (87.1%)	2,205 (86.0%)	0.220
N1	886 (11.7%)	360 (14.0%)		330 (12.9%)	360 (14.0%)	
**AJCC M stage**, ***n*** **(%)**						
M0	6,521 (86.3%)	2,068 (80.6%)	<0.001	2,091 (81.5%)	2,068 (80.6%)	0.412
M1	1,038 (13.7%)	497 (19.4%)		474 (18.5%)	497 (19.4%)	
**Treatment**, ***n*** **(%)**						
Only surgery	2,309 (30.5%)	699 (27.3%)	0.001	684 (26.7%)	699 (27.3%)	0.953
Only radiation	2,690 (35.6%)	844 (32.9%)		847 (33.0%)	844 (32.9%)	
Both surgery and radiation	741 (9.8%)	290 (11.3%)		287 (11.2%)	290 (11.3%)	
No surgery and radiation	1,819 (24.1%)	732 (28.5%)		747 (29.1%)	732 (28.5%)	
**PSA (ng/ml)**						
<10	3,371(44.6%)	1,032 (40.2%)	<0.001	1,063 (41.4%)	1,032 (40.2%)	0.675
≥10& <20	1,661 (22.0%)	559 (21.8%)		545 (21.2%)	559 (21.8%)	
≥20	2,527 (33.4%)	974 (38.0%)		957 (37.3%)	974 (38.0%)	
**Marital status**, ***n*** **(%)**						
Unmarried	2,019 (26.7%)	735 (28.7%)	0.056	705 (27.5%)	735 (28.7%)	0.351
Married	5,540 (73.3%)	1,830 (71.3%)		1,860 (72.5%)	1,830 (71.3%)	

*P < 0.05 was considered as statistically significant. PSM, propensity-score matching; NA, not available; IQR, interquartile range; The word “Unmarried” in marital status means unmarried, divorced, widowed, separated and never married, etc*.

### Effects of GP on Prognosis in the After-PSM Cohort

To compare the prognosis between the GP 4 + 5 and the GP 5 + 4 groups in the absence of effects from other covariates, a 1:1 ratio of the PSM process was performed to generate a cohort of 5,130 patients. The baseline characteristics were well-balanced (Table 1). In the matched groups, the GP 5 + 4 group had a poorer OS than the GP 4 + 5 group (5-year OS: 0.601 vs. 0.657, HR = 1.15, 95% CI: 1.07–1.24, *p* < 0.001; Table 2; Figure 2C). The GP 5 + 4 group was also associated with a higher cumulative incidence of CSM compared with the GP 4 + 5 group (5-year CIF: 0.282 vs. 0.230; SHR = 1.26, 95% CI: 1.15–1.39, *p* < 0.001; Table 3; Figure 2D).

**Table 2:** Univariate and multivariate Cox analyses of OS (*n* = 10,124).

	**5-year OS %**	**10-year OS %**	**Univariate analysis**			**Multivariate analysis**		
			**HR**	**95% CI**	***P*-value**	**HR**	**95% CI**	***P*-value**
**Age groups (y)**								
<72	0.761	0.580	Reference			Reference		
72–78	0.626	0.367	1.81	1.70–1.92	<0.001	1.64	1.54–1.75	<0.001
>78	0.357	0.094	4.07	3.78–4.39	<0.001	2.91	2.68–3.15	<0.001
**Race**								
Other	0.774	0.569	Reference			Reference		
Black	0.605	0.396	1.71	1.50–1.94	<0.001	1.52	1.34–1.74	<0.001
White	0.685	0.472	1.35	1.20–1.51	<0.001	1.37	1.22–1.54	<0.001
**AJCC T stage**								
T1/T2	0.657	0.435	Reference			NA		
T3/T4	0.728	0.534	0.766	0.722–0.813	<0.001	NA	NA	NA
**AJCC N stage**								
N0	0.700	0.486	Reference			Reference		
N1	0.542	0.342	1.58	1.47–1.71	<0.001	1.12	1.04–1.22	0.005
**AJCC M stage**								
M0	0.765	0.535	Reference			Reference		
M1	0.206	0.0900	5.22	4.90–5.57	<0.001	3.07	2.85–3.32	<0.001
**Surgery methods**								
No surgery and radiation	0.387	0.174	Reference			Reference		
Only surgery	0.800	0.639	0.237	0.219–0.255	<0.001	0.569	0.522–0.621	<0.001
Only radiation	0.749	0.499	0.355	0.333–0.379	<0.001	0.669	0.622–0.719	<0.001
Both surgery and radiation	0.818	0.604	0.260	0.233–0.289	<0.001	0.626	0.556–0.704	<0.001
**PSA (ng/ml)**								
<10	0.812	0.617	Reference			Reference		
≥10& <20	0.731	0.472	1.53	1.42–1.65	<0.001	1.27	1.18–1.37	<0.001
≥20	0.481	0.276	2.92	2.74–3.11	<0.001	1.61	1.50–1.73	<0.001
**Marital status**								
Unmarried	0.585	0.372	Reference			Reference		
Married	0.716	0.504	0.669	0.631–0.710	<0.001	0.803	0.756–0.852	<0.001
**Gleason score**								
4 + 5	0.707	0.492	Reference			Reference		
5 + 4	0.601	0.397	1.35	1.28–1.44	<0.001	1.26	1.18–1.34	<0.001
**After PSM (*****n*** **=** **5,130)**								
**Gleason score**								
4 + 5	0.657	0.440	Reference			NA		
5 + 4	0.601	0.397	1.15	1.07–1.24	<0.001	NA		

*OS, overall survival; HR, hazard ratio; CI, confidence interval; PSA, prostate-specific antigen; The word “Unmarried” in marital status means unmarried, divorced, widowed, separated and never married, etc*.

**Table 3:** Univariate and multivariate analysis for CSS using the competing risk model (*n* = 10,124).

	**Cumulative incidence function**			**Univariate analysis**			**Multivariate analysis**		
	**36-month**	**60-month**	**120-month**	**SHR**	**95% CI**	***P*-value**	**SHR**	**95% CI**	***P*-value**
**Age groups (y)**									
<72	0.130	0.198	0.305	Reference			Reference		
72–78	0.143	0.217	0.331	1.11	1.02–1.20	0.014	1.03	0.940–1.13	0.524
>78	0.198	0.295	0.437	1.58	1.42–1.76	<0.001	1.12	0.979–1.27	0.100
**Race**									
Other	0.101	0.155	0.242	Reference			Reference		
Black	0.177	0.266	0.398	1.83	1.55–2.17	<0.001	1.47	1.24–1.75	<0.001
White	0.138	0.209	0.321	1.39	1.20–1.62	<0.001	1.34	1.15–1.56	<0.001
**AJCC T stage**									
T1/T2	0.137	0.207	0.317	Reference			Reference		
T3/T4	0.149	0.225	0.342	1.10	1.02–1.18	0.010	1.14	1.05–1.25	0.003
**AJCC N stage**									
N0	0.124	0.190	0.295	Reference			Reference		
N1	0.262	0.383s	0.551	2.29	2.10–2.50	<0.001	1.25	1.12–1.40	<0.001
**AJCC M stage**									
M0	0.0908	0.147	0.243	Reference			Reference		
M1	0.458	0.642	0.833	6.44	5.94–6.98	<0.001	4.04	3.64–4.49	<0.001
**Surgery methods**									
No surgery and radiation	0.258	0.381	0.552	Reference			Reference		
Only surgery	0.0971	0.151	0.240	0.342	0.312–0.375	<0.001	0.711	0.629–0.805	<0.001
Only radiation	0.104	0.161	0.255	0.366	0.366–0.399	<0.001	0.726	0.654–0.807	<0.001
Both surgery and radiation	0.117	0.181	0.284	0.416	0.368–0.470	<0.001	0.828	0.709–0.967	0.017
**PSA (ng/ml)**									
<10	0.0823	0.129	0.206	Reference			Reference		
≥10& <20	0.113	0.176	0.277	1.40	1.27–1.55	<0.001	1.25	1.12–1.38	<0.001
≥20	0.237	0.352	0.517	3.15	2.91–3.41	<0.001	1.68	1.52–1.85	<0.001
**Marital status**									
Unmarried	0.169	0.254	0.383	Reference			Reference		
Married	0.130	0.198	0.305	0.755	0.700–0.814	<0.001	0.906	0.833–0.986	0.025
**Gleason score**									
4 + 5	0.126	0.193	0.297	Reference			Reference		
5 + 4	0.184	0.275	0.411	1.50	1.40–1.62	<0.001	1.38	1.27–1.50	<0.001
**After PSM (*****n*** **=** **5,130)**									
**Gleason score**									
4 + 5	0.157	0.230	0.339	Reference			NA		
5 + 4	0.194	0.282	0.407	1.26	1.15–1.39	<0.001	NA		

*SHR, subdistribution hazard ratio; CI, confidence interval; RN, radical nephroureterectomy; NSS, nephron-sparing surgery*.

### Independent Predictors on OS

**Figure 3:**
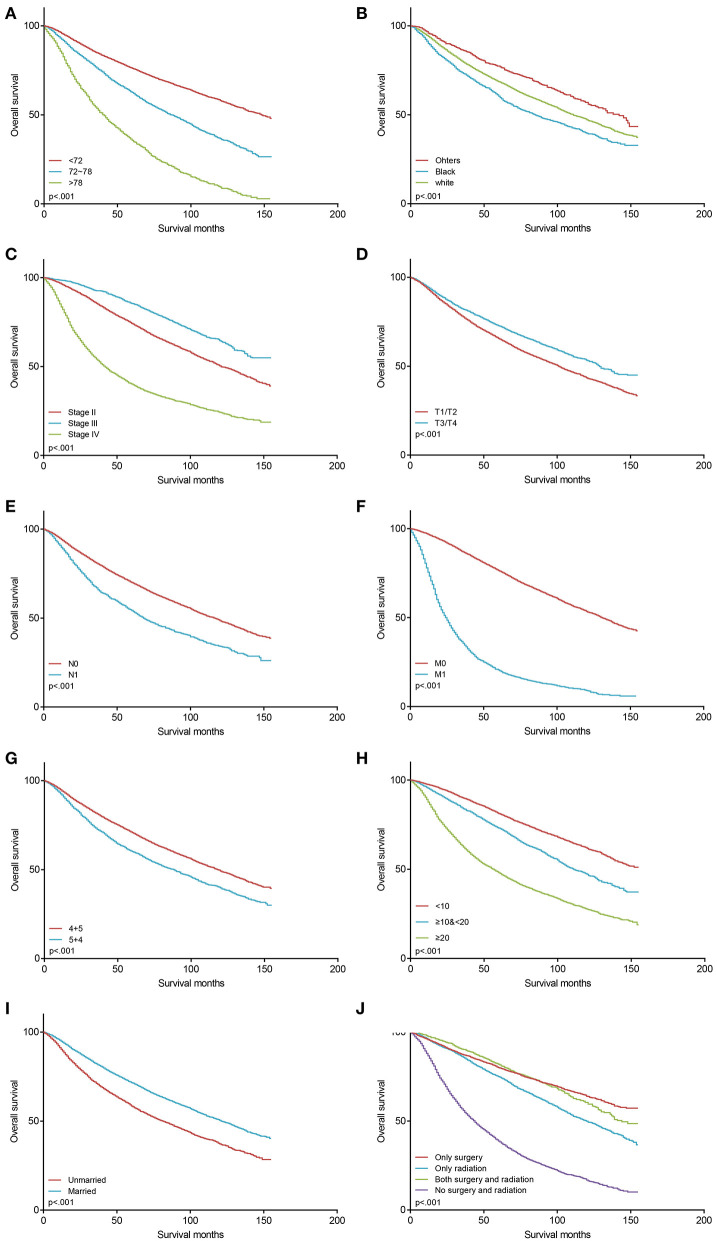
The Kaplan–Meier survival curves of OS before PSM (*n* = 10,124). **(A)** survival curves for age at diagnosis; **(B)** survival curves for race; **(C)** survival curves for AJCC stage; **(D)** survival curves for AJCC T stage; **(E)** survival curves for AJCC N stage; **(F)** survival curves for AJCC M stage; **(G)** survival curves for Gleason patterns; **(H)** survival curves for PSA levels; **(I)** survival curves for marital status; **(J)** survival curves for treatments. OS, overall survival; PSM, propensity score-matching; PSA, prostate-specific antigen.

The Kaplan-Meier curves stratified by covariates, which were age at diagnosis, race, AJCC stage, AJCC TNM stage, PSA levels, GP, marital status, and treatments, were shown in Figure 3. GP 4 + 5 was associated with a better OS than GP 5 + 4 (5-year OS: 0.707 vs. 0.601, *p* < 0.001; Figure 3G). The AJCC stage could be confirmed based on the AJCC TNM stage, GS, and PSA levels. Therefore, the AJCC stage was not included in the further analyses as a covariate. All the nine factors were associated with OS significantly in the univariate Cox analysis (Table 2), among which, GP 4 + 5 was still a protective factor of OS (HR = 1.35, 95% CI 1.28–1.44, *p* < 0.001). A forward stepwise multivariate Cox analysis confirmed that the eight variables, except the AJCC T stage, were independent predictors of OS (Table 2). After adjusting other covariates, GP 5 + 4 was still associated with a poorer OS than GP 4 + 5 (HR = 1.26, 95% CI 1.18–1.34, *p* < 0.001).

### Independent Predictors on CSS

**Figure 4:**
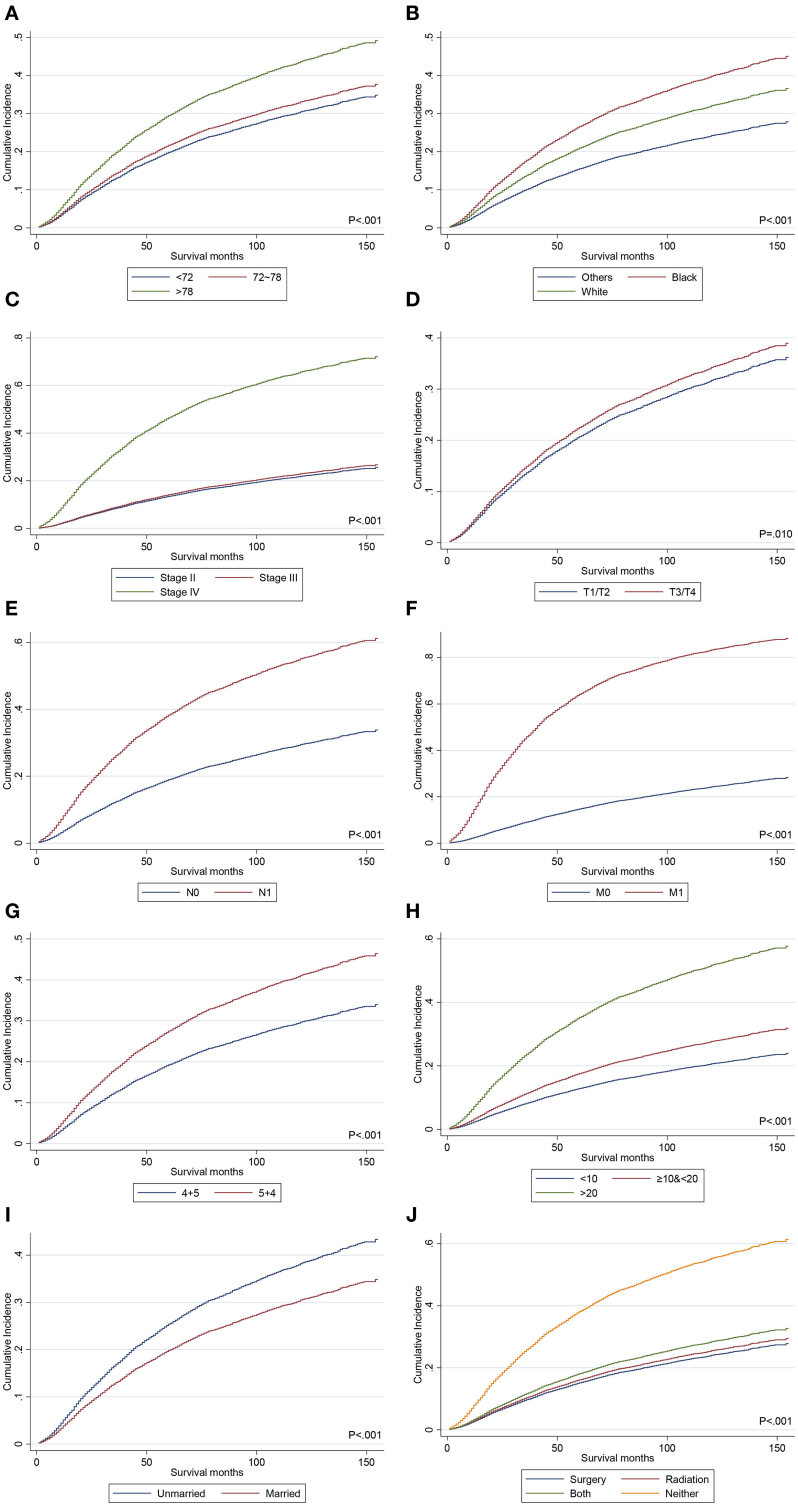
The CIF curves of CSM before PSM. **(A)** CIF curves for age at diagnosis; **(B)** CIF curves for race; **(C)** CIF curves for AJCC stage; **(D)** CIF curves for AJCC T stage; **(E)** CIF curves for AJCC N stage; **(F)** CIF curves for AJCC M stage; **(G)** CIF curves for Gleason patterns; **(H)** CIF curves for PSA levels; **(I)** CIF curves for marital status; **(J)** CIF curves for treatments. CIF, cumulative incidence function; CSM, cancer-specific mortality; PSM, propensity score-matching; PSA, prostate-specific antigen.

Deaths from non-PCa causes were regarded as competing events of cancer-specific mortality. Fine-Gray competing-risk regression models revealed that race, AJCC TNM stage, PSA levels, marital status, treatments, and GP were independent predictors for CSS in the univariate and multivariate analyses (Table 3, Figure 4). Specifically, GP 5 + 4 was associated with a poorer CSS compared with GP 4 + 5. The GP 4 + 5 group had a lower 3, 5, and 10-year CIF (0.126 vs. 0.184, 0.193 vs. 0.275, 0.297 vs. 0.411) than the GP 5 + 4 group. In the multivariate analysis, the GP 5 + 4 group had a higher risk of CSM than the GP 4 + 5 group (SHR = 1.38, 95% CI 1.27–1.50, *p* < 0.001). Although the AJCC T stage was not proven to be an independent predictor for OS in the multivariate Cox analyses, the higher T stage indicated higher risks of CSM in the multivariate competing-risk regression (SHR = 1.14, 95% CI 1.05–1.25, *p* = 0.003). Age at diagnosis was not proven to be significantly associated with CSS. Given that age at diagnosis was a powerful predictor for OS, we included it into the final multivariate competing-risk model.

## Discussion

The Gleason grading system was widely used since the 1960s (3) to stratify the risk for patients with prostate cancer, which is based on a five-histologic pattern system. GS, not exceeding 10, can be calculated by the sum of the most prevalent histologic pattern and the second most prevalent histologic pattern of prostate specimen. A higher GS indicates a higher degree of malignancy, acting as a robust predictor of progressions, metastasis, and survival. GS 9–10 was categorized as grade group 5 (6). However, different proportions of GP 5 and GP 4, which are 4 + 5 or 5 + 4, can both lead to GS 9. Previous studies reported that the dominant pattern in GS 7 PCa (4 + 3 vs. 3 + 4) provided a stronger power when predicting the prognosis (6, 15, 16). Similarly, this study aimed at comparing the prognosis between the GP 4 + 5 group and the GP 5 + 4 group.

It was reported that an increased proportion of Gleason pattern 5 had a trend to be associated with adverse outcomes (7, 17). There have been several published studies comparing the prognosis between GP 4 + 5 PCa and GP 5 + 4 PCa. Lim et al. published a study including patients with GP 4 + 5 (*N* = 58) and GP 5 + 4 (*N* = 22) tumors (8). GP 4 + 5 PCa was associated with a significantly lower percentage of lymph node involvement and a statistically significantly better biochemical recurrence-free survival compared with the biopsy of GP 5 + 4 tumors. Tilki et al. retrospected the follow-up information of 922 men with PCa, of whom, 295 (32.0%) were diagnosed with GP 5 + 4 PCa on biopsy and 627 (68.0%) were diagnosed with GP 4 + 5 on biopsy (9). They got the conclusion that GP 5 + 4 PCa was associated with a poorer CSS and a higher risk of metastasis compared with GP 4 + 5 PCa. Stroup et al. identified 634 patients with GS 8–10 PCa, of whom, 240 (38%) had tumors with a GP 5 component (i.e., GP 3 + 5, GP 5 + 3, GP 4 + 5, GP 5 + 4, or GP 5 + 5) and 394 (62%) had GP 4 + 4 tumors. The two groups showed no significant difference in the risk of biochemical recurrence (BCR). However, they discovered that PCa with GP 5 was associated with greater risks of metastasis, CSM, and ACM (18). Nanda et al. reported that PCa with GS 7 as well as tertiary grade 5 had a similar risk of BCR compared with PCa with GS 9–10. Meanwhile, GS 8 PCa was associated with a lower risk of BCR than GS 9–10 PCa (10). The results suggested that the highest or dominant GP of PCa determined the prognosis more powerfully. Meanwhile, it is worth noting that these mentioned studies were all limited by the sample size. The SEER database recorded follow-up information of millions of patients with cancer across America. There are enough subjects recorded in the SEER database to analyze.

PCa is more common in elderly men, who were under a variety of life-threatening risks other than tumors. Deaths from other causes act as competing events to CSM, whose appearances prevent the events of interest from happening (19). On this occasion, Kaplan–Meier methods and Cox analysis are not suitable for the analysis of CSS. Therefore, in this study, Kaplan–Meier methods and Cox analyses were used to confirm the variables affecting OS and Fine-Gray competing-risk models were used to confirm the variables affecting CSS. In the entire cohort (*N* = 10,124), PCa of GP 5 + 4 was associated with a poorer OS and CSS compared with PCa of GP 4 + 5. After balancing the baseline clinical variables by a 1:1 ratio of the PSM method, the negative effects of GP 5 + 4 on OS and CSS still existed compared with GP 4 + 5. Our results, consistent with previous studies (8–10, 18), revealed that the dominant GP was more prognostic.

We confirmed eight independent predictors for OS (Table 2) and nine for CSS (Table 3). Older age at diagnosis, black and white Americans, N1 stage, M1 stage, not accepting surgery and radiation therapy, higher PSA levels, GP 5 + 4, and unmarried status were associated with a poorer OS in the entire cohort (*N* = 10,124). Black and white Americans, N1 stage, M1 stage, not accepting surgery and radiation therapy, higher PSA levels, GP 5 + 4, unmarried status, and higher AJCC T stage predicted worse CSS. The covariates in the models were all easy to confirm or consistent with the currently accepted guidelines.

The PSA values were stratified into three levels, <10, ≥10& <20, and ≥20 ng/ml, consistent with the AJCC Cancer Staging Manual, Eighth Edition (2017) (6). The grouping of age at diagnosis was accomplished by X-tile (14). It is understandable that an older age at diagnosis was associated with a poorer OS in the multivariate Cox analyses. Unexpectedly, age at diagnosis was not an independent predictor for CSS in the multivariate competing-risk model. Considering the fact that age at diagnosis was a powerful independent covariate in the model for OS and previous studies (15, 20, 21), it was finally taken into the model for CSS. AJCC T stage was not included in the model for OS, but it was an independent covariate for CSS. The negative effects of marital status on the prognosis of patients with PCa were confirmed in some published studies (22). As a social exterior factor, an unmarried status was associated with a poorer prognosis in this study, which may be explained that unmarried people were short of social support in general (23). It has been reported that African Americans had a higher incidence and mortality of PCa (1, 24). In this study, ethnicity did work as an independent predictor in the models.

To our knowledge, this study is the first population-based investigation about the prognosis of patients with GS 9 PCa using data from the SEER registries. The results confirmed the negative effects of GP 5 + 4 on the prognoses compared with GP 4 + 5.

There are still some limitations to this study. Firstly, the follow-up information in this study was limited by the records from the SEER database. Some variables about the treatments and disease progression were not recorded detailedly by the SEER registries, such as the PSA values over time, endocrinotherapy, metastatic sites, evidence of local recurrence, and so on. Therefore, it was difficult to determine the BCR and progression-free survival of each patient. In addition, treatments were roughly categorized into four types. Other basic information about the patients, such as hemoglobin levels, body mass index, and smoking history, was hard to confirm. Secondly, there was a lack of external data to validate the negative effects of GP 5 + 4 on the prognoses. We, coauthors, call on urologists to confirm the results in this study with the follow-up data of their own clinical centers. Thirdly, this is a retrospective study. Potential bias still existed even after the PSM or multivariate analysis.

## Conclusion

This population-based study reported the negative effects of GP 5 + 4 on the OS and CSS compared with GP 4 + 5, after eliminating the effects from other variables.

## Data Availability Statement

Publicly available datasets were analyzed in this study. This data can be found at: https://seer.cancer.gov/data/.

## Ethics Statement

Ethical review and approval was not required for the study on human participants in accordance with the local legislation and institutional requirements. Written informed consent for participation was not required for this study in accordance with the national legislation and the institutional requirements.

## Author Contributions

JQ and JZ: conceptualization. JQ and DC: data curation and original draft writing. JQ, DC, and ZW: formal analysis. KG: funding acquisition. KG, YG, and LC: methodology, draft review, and revision. KG and LC: project administration and supervision. All authors contributed to the article and approved the submitted version.

## Conflict of Interest

The authors declare that the research was conducted in the absence of any commercial or financial relationships that could be construed as a potential conflict of interest.
